# A Metal Coordination-Based Supramolecular Elastomer with Shape Memory-Assisted Self-Healing Effect

**DOI:** 10.3390/polym14224879

**Published:** 2022-11-12

**Authors:** Fang Xie, Zhongxin Ping, Wanting Xu, Fenghua Zhang, Yuzhen Dong, Lianjie Li, Chengsen Zhang, Xiaobo Gong

**Affiliations:** 1 School of Materials Science and Engineering, Harbin Institute of Technology at Weihai, Weihai 264209, China; 2 National Key Laboratory of Science and Technology on Advanced Composites in Special Environments, Harbin Institute of Technology, Harbin 150080, China; 3 School of Naval Architecture and Ocean Engineering, Harbin Institute of Technology at Weihai, Weihai 264209, China

**Keywords:** self-healing, polybutadiene, metal coordination, shape memory, oxidized carbon nano-onions

## Abstract

Rubber materials are widely used in aerospace, automotive, smart devices and artificial skin. It is significant to address the aging susceptibility of conventional vulcanized rubber and to impart it rapid self-healing performance for destructive crack damage. Herein, a novel supramolecular rubber elastomer is prepared by introducing metal coordination between carboxyl-terminated polybutadiene and polystyrene-vinylpyridine copolymer. Based on the metal coordination interaction, the elastomer exhibits shape memory and self-healing properties. Moreover, a rapid closure-repair process of destructive cracks is achieved by presetting temporary shapes. This shape memory-assisted self-repair model is shown to be an effective means for rapid repair of severe cracks. An approach to enhance the mechanical and self-healing properties of elastomer was demonstrated by adding appropriate amounts of oxidized carbon nano-onions (O-CNO) into the system. The tensile strength of the elastomer with an O-CNOs content of 0.5 wt% was restored to 83 ± 10% of the original sample after being repaired at 85 °C for 6 h. This study confirms that metal coordination interaction is an effective method for designing shape memory self-healing rubber elastomer. The shape memory-assisted self-healing effect provides a reference for the rapid self-repairing of severe cracks.

## 1. Introduction

Rubber has been widely used in aerospace, automotive, smart devices and artificial skin due to its excellent performance due to its high elasticity and high wear resistance. The traditional rubber generally needed to undergo vulcanization is processed to cross-link the raw rubber molecules to form a 3D network to optimize its performance. However, a stable and an irreversible covalent cross-linked structure makes the rubber difficult to recycle once it is aged. Therefore, developing a new elastomer molecular network construction approach to manufacture rubber materials with excellent mechanical properties and self-healing efficiency remains a challenge [1,2,3].

Supramolecular interactions include hydrogen bonding interaction, metal coordination interaction, π–π stacking interaction, host–guest interaction and other intermolecular interactions [4,5]. Urban et al. [6] pointed out that supramolecular interactions have faster polymer network reconstruction speed and sensitivity compared with covalent bonds. Moreover, compared with other supramolecular interactions, such as hydrophobic interaction and hydrogen bond interaction, metal coordination has strong interaction forces, strict directivity, abundant ligands and it is easy to modify, thus having broad designability and diverse functions [7]. The supramolecular polymers based on metal coordination can simultaneously obtain the excellent properties of polymers and metal complexes [8,9]. Metal coordination will provide the possibility for the design of new self-healing shape memory elastomers.

Liu [10] et al. combined hydrogen bonding and Zn(II)-triazole coordination to prepare a high-performance elastomer with a dual dynamic network. The tensile strength of the elastomer recovered to 74% of that of the original sample (15.5 MPa) after healing at 80 °C for 24 h. Zhao et al. [11] developed dual-programmable shape-memory organohydrogels with supramolecular heterogeneous networks by utilizing Fe(III)-carboxylate coordination. This organohydrogels exhibits excellent self-healing properties and hierarchical shape morphing performance. Li et al. [12] prepared an elastomer with high stretchability and self-healing ability based on strong pyridine-Fe(III) and weak formamide-Fe(III) coordination. The healing efficiency of this elastomer was 90 ± 3% after 48 h of healing at room temperature. Wu et al. [13] introduced strong metal coordination bonds and weak hydrogen bonds into the polymer network to prepare a supramolecular elastomer with good mechanical properties and excellent self-healing ability, with a high self-healing efficiency of 90%.

However, the properties of these self-healing elastomers are relatively single, generally showing good mechanical properties and self-healing properties. Here, we consider introducing metal coordination bonds into the block copolymer network, using the microphase separation properties of block copolymers and the metal coordination of supramolecular polymers to make the polymers form metal coordination block copolymer micelles [14,15,16,17]. Dynamic coordination interaction is used to ensure the spontaneous progress of self-healing and the aggregated structure of micelles is used to provide the nodes and networks required for shape memory polymers, while shape memory effect can assist self-healing behavior and further improve self-repair efficiency or shorten the time in the same case.

Moreover, carbon nano-onions (CNOs) with unique structural properties are typical 0D nanomaterials with high porosity, high electrical conductivity, and electrochemical stability [18,19,20]. Surface-functionalized CNOs not only possess good chemical/mechanical stability, high electrical conductivity, and tunable surface structure [21,22,23] but also have good dispersibility and barely agglomeration [24,25]. Compared with other widely studied carbon materials, the functionalized CNOs exhibit higher reactivity and solubility. The oxidized CNOs (O-CNOs) have abundant carboxyl groups, which may provide a strong interfacial force between O-CNOs and polymer subjects so as to form composites with better mechanical properties [26,27]. Additionally, O-CNOs with abundant carboxyl groups can increase the content of hydrogen bonds in the complexes to form strong interfacial interactions, strengthening the mechanical properties, while providing an extra interfacial driving force for the repairing the broken interfaces [28]. For this study, O-CNOs are prepared by referring to the previously reported method [29], and their effects on the mechanical properties and self-healing properties of the system are investigated.

In this paper, a self-healing polybutadiene-polystyrene-vinylpyridine elastomer based on metal coordination bond interaction [Zn(II)-carboxyl group and Zn(II)-pyridine] was prepared using polybutadiene rubber as the base material. An appropriate amount of O-CNOs was added to the elastomer to enhance its mechanical properties and self-healing ability. The well-dispersed O-CNOs reduced the intermolecular chain interaction forces, and the polymer chain mobility was enhanced, thus improving the self-healing ability. Moreover, the functionalized O-CNOs provide more abundant carboxyl groups, which enhanced the internal hydrogen bonding to improve the mechanical properties. The shape memory-assisted self-repair model was proposed as an effective means for rapid repair of large cracks. This multifunctional material integrating shape memory, self-healing, and shape-memory-assisted self-repairing properties will play a unique role in artificial muscles, artificial skin, intelligent control, sensing and other fields.

## 2. Materials and Methods

### 2.1. Materials

Carboxyl-terminated polybutadiene (PB-COOH, average molecular weight of 4200 g/mol) was purchased from Tianjin Xiens Biochemical Technology Co., Ltd. (Tianjin, China). The polystyrene-vinylpyridine (PS-b-P4VP) was purchased from Scientific Polymer Products; molecular weight was 400,000, and the content of pyridine functional group was 50 mol%. Toluene, ethanol and zinc acetate dihydrate were purchased from Aladdin Chemical Reagent Company. Carbon nano-onions (CNOs, diameters: 5–7 nm) were purchased from Nanjing Mingchang New Material Technology Co., Ltd. (Nanjing, China). All chemicals were commercially available and used as received.

### 2.2. Preparation of Polybutadiene-Polystyrene-Vinylpyridine Elastomer

**Figure 1:**
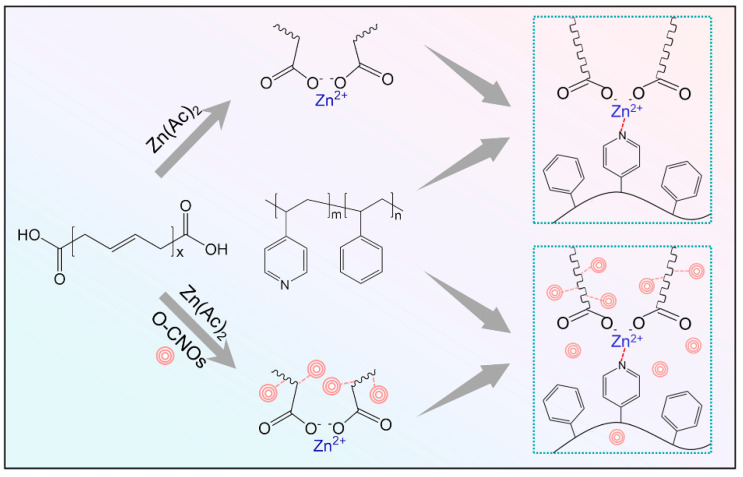
Schematic diagram of the preparation process of self-healing polybutadiene rubber composite without or with O-CNOs.

The compositions of all samples are listed in Table 1, and the samples were prepared according to the following procedure (Figure 1). Firstly, zinc acetate was dissolved in deionized water and methanol mixture with volume ratio of 1:1, and PB-COOH was dissolved in toluene. The above two solutions were mixed and stirred for 48 h and then vacuum-dried at 50 °C for 24 h to remove small molecular solvents, such as water, methanol, acetic acid and toluene, to obtain zinc ionized carboxyl-terminated polybutadiene (PB-COOZn). PB-COOZn and PS-b-P4VP were respectively dissolved in toluene solution; the two solutions were mixed and magnetically stirred for 48 h. The mixture was dried in an oven at 50 °C for 72 h to remove most of the solvent and then dried in a vacuum drying oven at 50 °C for 24 h to remove all solvents to obtain the metal ion complex of PB-COOZn and PS-b-P4VP. The samples with the molar ratios of Zn^2+^, COO^-^ and VP of 1:2:2, 1:2:4 and 1:2:8 were denoted as R1, R2 and R3, respectively.

The CNOs and concentrated sulfuric acid were mixed to prepare a suspension; under the condition of constant stirring, concentrated sulfuric acid and concentrated nitric acid mixture with volume ratio of 3:5 was added to the above suspension and then cooled in an ice-water bath for 30 min. The mixture was then heated at 80 °C for 30 min to obtain a dispersion. Distilled water was added to the dispersion to dilute the dispersion; the dilution was centrifuged at 7500 rpm after standing for 12 h, and the dilution and centrifugation process was repeated twice. The black solid was collected to obtain O-CNOs powder.

O-CNOs powder and zinc acetate were dispersed in water/ethanol solution. PB-COOH and zinc acetate were dissolved in toluene solution. Mix the above two solutions well and let stand. After the solution was separated, PS-b-P4VP/toluene solution was added to it, and finally a gray mixed solution was formed. Use a rotary evaporator to remove most of the solvent from the grey mixed solution. The mixture was dried in a vacuum oven for 24 h to remove residual solvent, and a gray-brown translucent composite material was obtained. The molar ratio of Zn^2+^, COO^−^ and VP in the prepared composite samples was 1:2:4, which were denoted as R2-0.5%, R2-1% and R2-2%, respectively, according to the mass ratio of their O-CNOs.

### 2.3. Characterization

Chemical bonds and functional groups of the samples were detected by Fourier Transform Infrared Spectroscopy (FT-IR) instrument (Nicolet 380, Thermal Electron, Waltham, MA, USA). The number of scans was 32 times/s. The test range was 400–4000 cm^−1^, and the transmission mode was used.

The molecular structure of the material was characterized by the laser microscope Raman spectrometer (Renishaw inVia, British). The wavelength was 532 nm, and the light intensity was 10%.

The thermal properties of the samples were measured with a Netzsch Instruments FTA449F5 differential scanning calorimetry (DSC) from 40 to 240 °C using a heating and cooling rate of 10 °C min^−1^ and an argon atmosphere. The data reported are from measurements made after first preheating the samples to 320 °C and then cooling them to 40 °C to eliminate the influence of the prior thermal history.

The quasi-static tensile test was performed on a Instron 5967 universal tensile testing machine with a stretching rate of 10 mm min^−1^. The tensile test uses dumbbell-shaped standard samples, and each specimen is tested more than five times in the same test environment to obtain the average value.

Cyclic tensile tests were performed on the samples at room temperature, the specimens were stretched to 50% strain, fully unloaded, and so on, and the hysteresis curves were measured at different cycles. The dissipated energy Δ*W* of the elastic body is obtained by calculating the integral area enclosed by the hysteresis curve by the following formula:

(1) $$\Delta W=\int_{load}\sigma d\varepsilon -\int_{unload}\sigma d\varepsilon$$

Analysis of self-healing performance: The wafer sample was cut into two semicircles with a blade and then healed at a certain temperature. The morphology of the sample before and after repair was observed by an optical digital microscope (DSX510, OLYMPUS, Tokyo, Japan).

Dumbbell-shaped elastomer samples were cut in half from the middle with a blade; the sections were aligned at room temperature, pressed gently for 1 min and then placed in a vacuum drying oven at room temperature and 85 °C for healing. After the repaired samples were taken out from the vacuum drying box, they were cooled to room temperature and then subjected to stress–strain testing at a tensile rate of 10 mm × min^−1^. The self-healing efficiency was quantitatively evaluated according to the ratio of the tensile strength and elongation at break of the healed material to the original material, respectively.

## 3. Results and Discussion

### 3.1. Molecular Structure of the Composite

**Figure 2:**
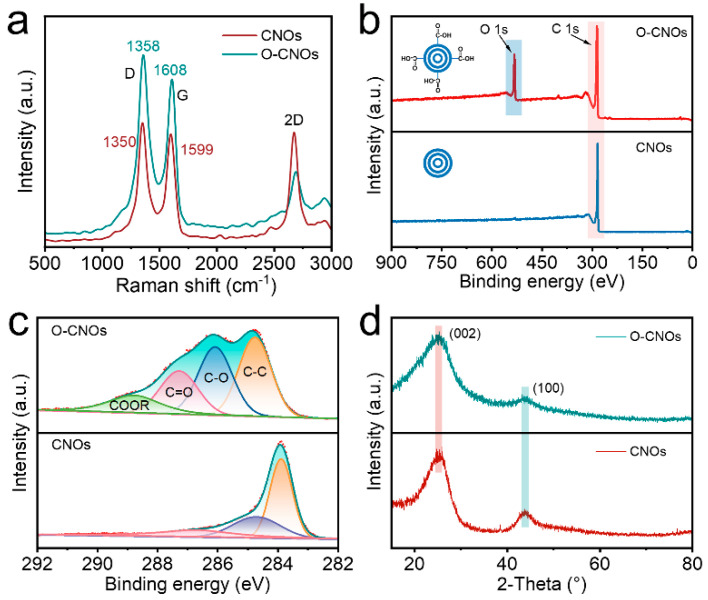
(**a**) Raman spectra of CNOs and O-CNOs obtained at oxidation time of 0.5 h. XPS analysis of all tested samples (**b**) and peak fitting of C 1s at high resolution (**c**). (**d**) XRD spectra of CNOs and O-CNOs.

The Raman spectras of CNOs and O-CNOs obtained at oxidation time of 0.5 h are shown in Figure 2. The D band and G band of the oxidized CNOs shifted to higher wavenumbers from 1350 cm^−1^, 1599 cm^−1^ to 1358 cm^−1^, 1608 cm^−1^, respectively. The ratio of intensities of the D and G bands (R = I_D_/I_G_) directly were related to the oxidation degree of the CNOs [28]. R of the pristine CNO was higher than that of O-CNO, which confirmed that CNOs had been successfully oxidized to O-CNOs. XPS results indicated that there were oxygen-containing functional groups on the surface of O-CNOs. The survey XPS spectra of the two samples (Figure 2b) revealed that there was a strong O 1S peak in O-CNOs compared with the original CNOs. This peak was largely attributed to the production of a large number of oxygen-containing functional groups on the acidified O-CNOs. Further analysis of the high-resolution C 1s peak (Figure 2c) showed that the surface of O-CNOs contains a mixture of carboxyl group (COOR), hydroxyl group (CO) and carbonyl (C=O). Note that the weak carbonyl peak observed in the C 1s spectrum of CNOs can be attributed to the adsorption of CO_2_ by the powder sample. The crystallinity of the CNOs and O-CNOs were studied by XRD (Figure 2d). The peaks at 2θ = 26° and 44° can be associated with the (002) and (100) diffractions of the hexagonal graphite structure. For O-CNOs, the peak intensity at 2θ = 44° was decreased slightly, which may be due to the increase of lattice defects after oxidation.

**Figure 3:**
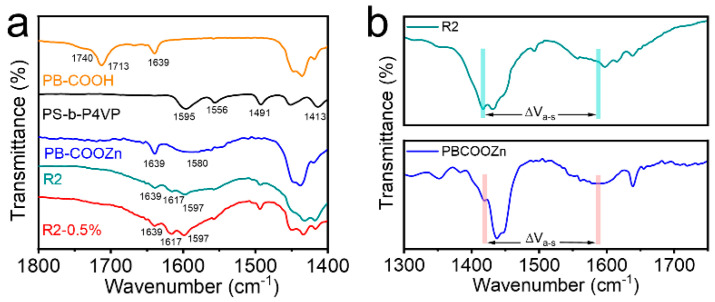
(**a**) FTIR spectra for component polymers and their blends. (**b**) The frequency separations (Δv_a-s_) between the symmetric and asymmetric stretching modes of COO^−^ in R2 and PBCOOZn.

As shown in Figure 3a, FTIR spectra was used to elucidate the successful construction of the dynamic network structure of polybutadiene composite rubber based on metal coordination. The comparison of the FTIR spectra of the original material and the sample is shown in Figure 3. The peaks of PB-COOH at 1639 cm^−1^, 1713 cm^−1^ and 1740 cm^−1^ corresponded to the olefin C=C stretching vibration, the hydrogen bond dimer, carboxyl groups and the carbonyl group (C=O) of free carboxyl groups, respectively. It can be seen that the peak of PB-COOZn at 1740 cm^−1^ disappeared, instead, a broader peak appeared at 1580 cm^−1^, which confirmed the successful bonding of PB-COOH to Zn^2+^. For PS-b-P4VP, the peaks at 1413 cm^−1^, 1491 cm^−1^, 1556 cm^−1^, and 1595 cm^−1^ corresponded to the aromatic vibration of the pyridine ring, and the peak at 1595 cm^−1^ corresponded to the carbon-nitrogen bond (C-N) vibration. The peak of R2 and R2-0.5% appear at 1617 cm^−1^ next to 1595 cm^−1^, revealing the complexation of Zn^2+^ with the N atom of pyridine on PS-b-P4VP [30]. The broad peak near 1580 cm^−1^ still existed, indicating that the complexation of Zn^2+^ with the carboxyl groups on the carboxyl-terminated polybutadiene was not disrupted. There was a clear relationship between the v_asym(COO_^−^_)_ and v_sym(COO_^−^_)_ bands in the FTIR spectra (region of 1300–1750 cm^−1^) and the metal-carboxylate coordination type. The separation of the bands (Δv_a-s_ = v_asym_ − v_sym_) can indicate of the structure of carboxylate [31]. The value for Δv_a-s_ for Zn^2+^ was almost equal than that of Na^+^, revealing that coordination mode in PB-COOZn was bidentate bridging (Figure 3b) [32].

### 3.2. Thermodynamic Property

**Figure 4:**
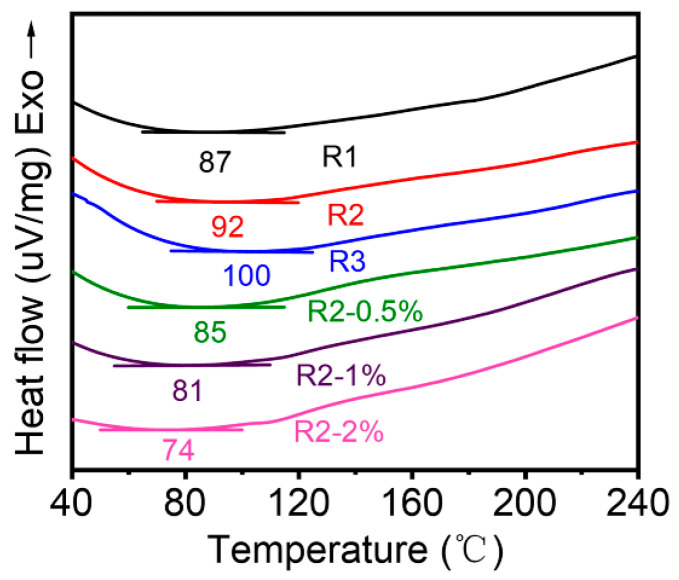
DSC curves of component polymers.

As shown in Figure 4 and Table 2, the DSC curves were used to reveal the thermodynamic properties of the samples. The T_g_ values of R1, R2 and R3 were 87 °C, 92 °C and 100 °C, respectively, indicating that more effective metal coordination bonds were formed with the increase of PS-b-P4VP content in the system, which led to the thermodynamic transformation tending to a higher temperature. However, the T_g_ values of the samples gradually decreased as the content of O-CNOs increased. This may be due to the poor interfacial interaction between PS-b-P4VP and O-CNOs in the system resulting in the formation of extra free volume between filler and matrix. The polymer chains near the interface optionally transfer into this free volume, resulting in increased chain mobility and decreased T_g_ value. Previous studies by BalajiKrishnakumar et al. [33] and GaniuB.Olowojoba et al. [34] showed that the T_g_ value gradually decreased with the increase of graphene oxide added to the epoxy resin system, and the same mechanism may play a role here. Meanwhile, since the self-healing performance of the material was closely related to the dynamic reversibility of metal coordination bonds, the reduction of T_g_ was beneficial to the self-healing property at low temperature.

### 3.3. Mechanical Property

**Figure 5:**
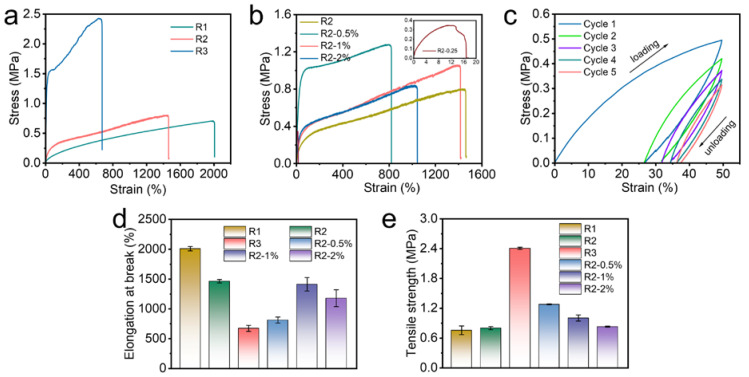
Mechanical properties of the samples. (**a**,**b**) Stress–strain curves for samples. (**c**) Continuous cyclic tensile loading–unloading curves at 50% strain without resting time between each cycle. (**d**,**e**) Elongation at break and Tensile strength of the samples.

Figure 5 shows the mechanical properties of various samples. With the increase of VP content, the tensile strength of the samples gradually increased from 0.76 MPa ± 0.09 MPa (R1) to 2.41 MPa ± 0.02 MPa (R3), and the elongation at break decreased gradually from 2009 ± 36% (R1) to 674 ± 50% (R3) (Figure 5a,d,e). Since the coordination number of Zn^2+^ was 4 [35,36], when the molar ratio of Zn^2+^:COO^−^:VP was 1:2:2, the molar ratio of Zn^2+^ to ligand reached the stoichiometric equivalent, and the tensile strength should reach the theoretical maximum value at this time. However, there will be uncoordinated free ligands in the system due to the steric hindrance and high viscosity of rubber. Therefore, higher molar ratios were required to ensure complete complexation and better mechanical properties.

With the increase of O-CNOs content, the tensile strength of the composites gradually decreased from 1.28 ± 0.01 MPa (R2-0.5%) to 0.83 ± 0.01 MPa (R2-2%) (Figure 5b,d,e). However, the tensile strengths of the samples added with O-CNOs (R2-0.5%, R2-1% and R2-2%) were consistently higher than the original samples (R2). However, the mechanical properties of R2-0.25% decreased sharply and were even lower than the R2. This appearance could be attributed to the formation of inhomogeneous defect sites in the composite due to the low content of OCNOs, which led to the decrease of the overall mechanical properties. This indicated that appropriate doping of O-CNOs can improve the mechanical properties of the composites through the interaction between O-CNOs and polymer segments, while excessive doping of O-CNOs may aggregate, which can create defects in the system, resulting in the poor mechanical properties of the composites.

**Figure 6:**
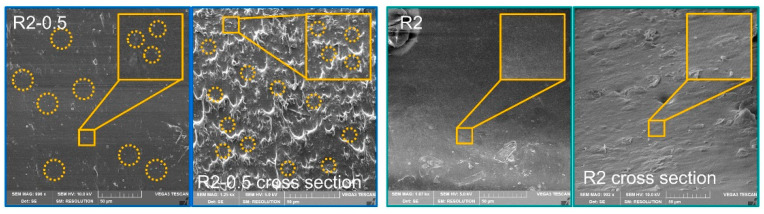
SEM images of R2-0.5 and R2 in surface and cross section mode.

The scanning electron microscope (SEM) was used to study the dispersion of O-CNOs in composite to illustrate the good mechanical properties. As shown in Figure 6, compared with R2, the uniformly dispersed O-CNOs in the surface and cross-section can be clearly observed in SEM images of R2-0.5. The uniform dispersion of O-CNOs was the guarantee to improve the mechanical properties of the composites.

**Figure 7:**
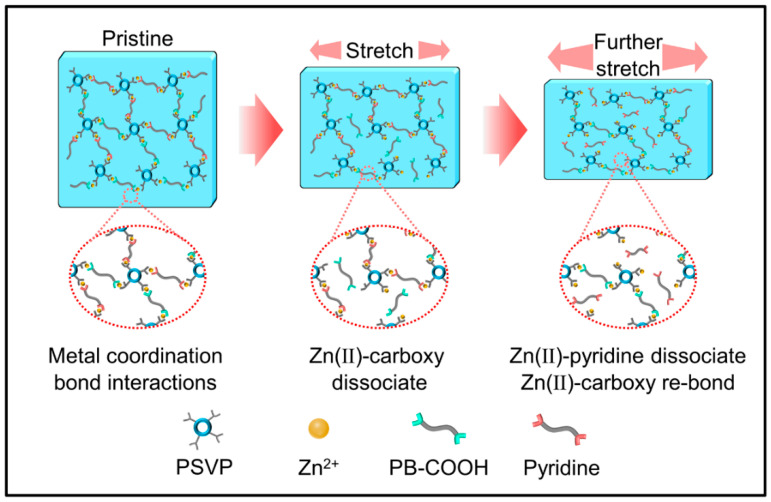
Schematic illustration of the self-healing mechanism.

Figure 5c shows the cyclic tensile loading–unloading curves of R2-0.5% at room temperature. The gradual reduction of the hysteresis loop with the progress of cyclic stretching proved the dynamic properties of metal coordination bonds. The bond energy of the coordination bond between the carboxyl group and Zn^2+^ was 104–134 kJ/mol [37], and that of between pyridine and Zn^2+^ was approximately 250 kJ/mol [38]. The Zn(II)-carboxy coordination bond with lower energy was broken first, and the Zn(II)-pyridine coordination bond with higher energy was broken later (Figure 7). The dynamic reversibility of the metal coordination bonds enabled the continuous breaking and reformation of the two coordination bonds (Zn(II)-carboxyl and Zn(II)-pyridine) during the stretching process and led to the folding and sliding of the polymer chain, which dissipated a mass of energy, resulting in the composite with superior tensile strength and toughness.

### 3.4. Self-Healing Ability

**Figure 8:**
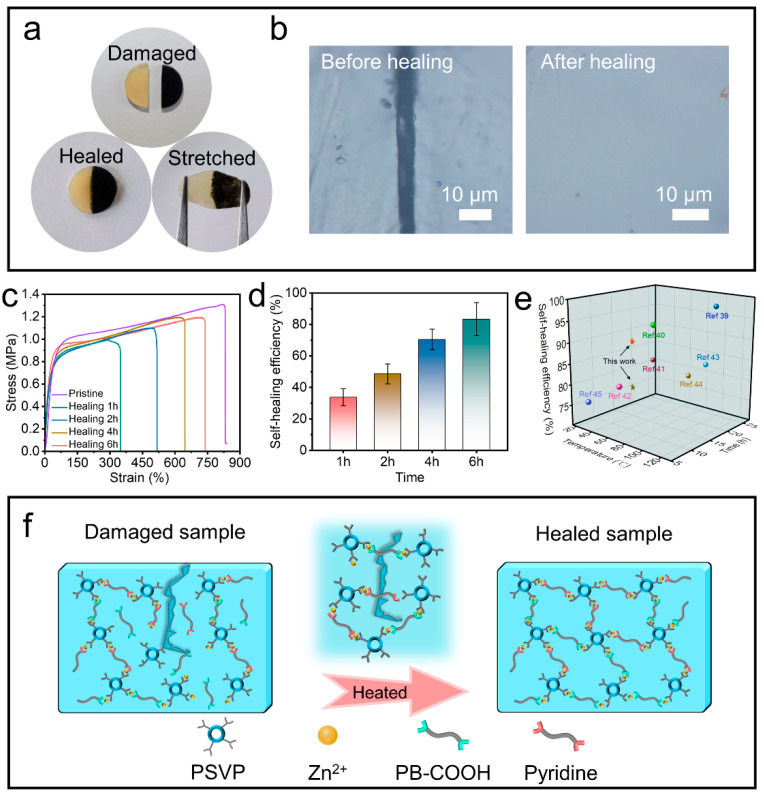
(**a**) Optical images of cut, healed and stretching R2/R2-0.5% specimen. (**b**) Microscope image of the surface of the sample. (**c**) Typical stress–strain curves with different healing times. (**d**) Corresponding healing efficiency of the R2-0.5% at different times. (**e**) Comparison of self-repairing performance using this work with previous reports [39,40,41,42,43,44,45]. (**f**) The proposed self-healing mechanism of R2-0.5%.

To investigate the self-healing properties of the R2-0.5%, the original composite was cut into two separate samples using a blade. The two sections formed after cutting were aligned and pressed gently for 1 min, which were transferred to a vacuum drying oven at 85 °C for healing. It can be seen that the fracture interface of the composite was completely healed and the crack disappeared (Figure 8a,b). Likewise, the same cutting-aligning-heat treatment was performed on dumbbell-shaped elastomer to achieve self-repairing behavior. The self-healed dumbbell-shaped composite was taken out and cooled to room temperature, and then subjected to stress–strain testing at a tensile rate of 10 mm min^−1^. Figure 8c shows the stress–strain curves and self-repairing efficiency of the composite after heat treatment at 85 °C for 1 h, 2 h, 4 h and 6 h, respectively. The results showed that the tensile strength and elongation at break of the composite gradually increased with the extension of heat treatment time and were more and more close to the original state. Note that the self-healing efficiency of the composite can reach 83 ± 10% after 6 h of heat treatment (Figure 8d). Compared with other advanced self-healing materials reported so far, the metal-ligand-based self-healing R2-0.5% has better self-healing properties (Figure 8e).

The essence of the excellent self-healing ability of composite was further explained by the supramolecular interaction. Figure 8f shows the mechanism of self-healing effect. When the composite was damaged, the two metal coordination bonds [Zn(II)-carboxyl and Zn(II)-pyridine] at the fractured interface were broken. The mobility of the polymer chains was greatly enhanced and accompanied by a reversible decrease in viscosity during the heat treatment at 85 °C, which promoted the rearrangement of the chains. Meanwhile, when the fractured interfaces were in contact with each other, the polymer chains diffused into each other and were accompanied by the reformation of two metal coordination bonds, resulting in the healing of the fractured interfaces. Additionally, both higher healing temperature and longer healing time endowed the polymer chains with better fluidity, resulting in superior self-healing ability and mechanical properties. Note that the O-CNOs as fillers reduced the T_g_ value of the R2 and thus enhanced the mobility of the polymer chains, which improved the self-healing properties of composite.

**Figure 9:**
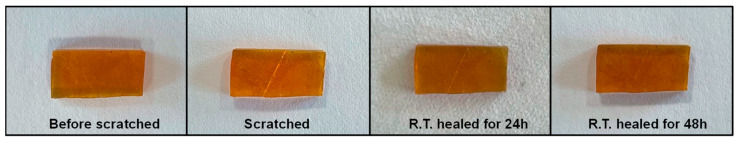
Self-healing effect occurred at room temperature (R.T.).

Note that the self-healing effect of R2 and R2-0.5 can also occur at room temperature. As shown in Figure 9, after 48 h of healing process at room temperature, the scratched incision on the both sample surfaces were bonded and only minor cracks were observed, demonstrating the great potential of metal coordination bonds with dynamic reversibility in room temperature self-repairing (Figure S1, Supporting Information). Moreover, R2-0.5% had a faster healing rate, which can be attributed to the addition of O-CNOs that reduced the T_g_ of the composite and enhanced the polymer chain flowability.

**Figure 10:**
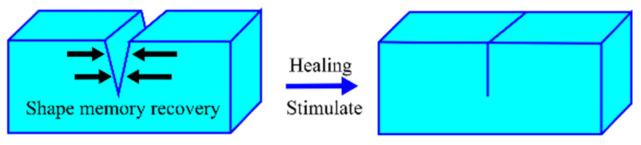
Schematic illustration of the shape memory assisted self-healing process.

Healing of polymeric materials included crack closure and crack healing. Crack closure was essential for subsequent crack healing. The damage of materials was often accompanied by large deformations or cracks inside the materials in practical applications, which made it difficult for the material to self-repair without external force intervention. To address this challenge, the shape memory effect was proposed to rapidly close the crack to facilitate the self-healing process (Figure 10). Herein, a series of novel supramolecular elastomers were prepared by introducing metal coordination between carboxyl-terminated polybutadiene and polystyrene-vinylpyridine copolymer. Under the synergistic effect of metal coordination and microphase separation of supramolecular polymers, the polystyrene-vinylpyridine aggregates can act as a network of nodes that define the permanent shape, the carboxyl-terminated polybutadiene aggregates can act as switching segments for fixing the temporary shape, and the Zn^2+^ acted as bridges to connect them, which allowed the system to generate shape memory effect.

Before that, the shape fixation rate and recovery rate before and after the addition of O-CNOs were calculated by testing the length of the original sample, the sample stretched to the elongation state, and the sample with shape recovery. The shape fixation rates of R2 and R2-0.5 were ~96% and ~93%, respectively, while their shape recovery rates were ~75% and ~64%, respectively. The addition of O-CNOs slightly led to the decrease of shape fixation and recovery rate of the composites, which may be attributed to the friction resistance produced by O-CNOs.

**Figure 11:**
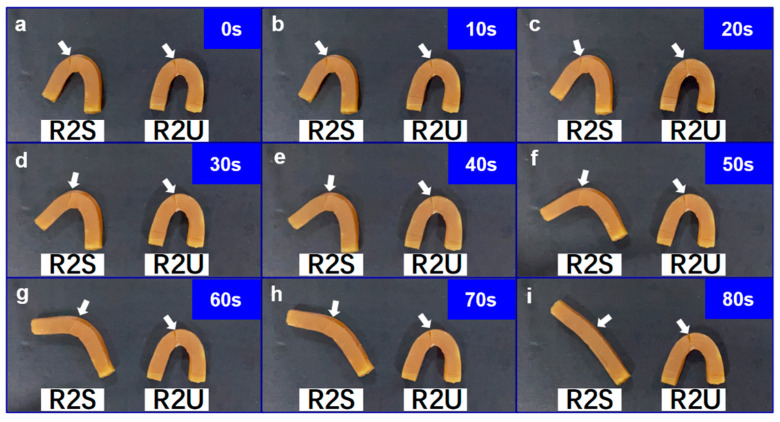
**Snapshots** of the shape memory assisted self-healing process. (**a**) 0s, (**b**)10s, (**c**) 20s, (**d**) 30s, (**e**) 40s, (**f**) 50s, (**g**) 60s, (**h**) 70s, (**i**) 80s.

Specifically, two samples with the same ratio as R2 were casted as U-shape and rectangular strip, and denoted as R2U and R2S, respectively. The rectangular strip sample R2S would endure a shape change and recovery process during which R2S was firstly bent into U-shape after being thoroughly heated at 60 °C and then cooled to fix the temporary U-shape. R2U and R2S, both in the U-shape state, were then scratched with a blade and simultaneously placed in a temperature environment of 60 °C (Figure 11a). As shown in Figure 11b–i, the incision on the sample R2S was quickly closed within 30 s, and the incision was completely healed within 80 s, while the incision of the R2U was not closed without external force intervention, and it was difficult to start its self-healing process. These results show that the prepared samples have outstanding shape memory assisted self-healing behavior. The energy stored by the temporarily state of shape memory process can quickly drive the crack closure, promote the start of self-healing process. This shape memory assisted self-healing performance can generally shorten the time required for self-healing and improve the self-repair efficiency. The similar shape memory-assisted self-healing performance of R2-0.5 was studied as mentioned above. As shown in Figure S2, U shaped R2-0.5 showed a good shape memory-assisted self-repair performance, which indicated that adding O-CNOs not only enhanced the mechanical properties and self-healing properties but also maintained the shape memory-assisted self-repairing performance.

## 4. Conclusions

In this paper, the elastomer with good shape memory effect, self-healing ability and mechanical properties was designed by introducing metal coordination and nano-filler into the polybutadiene rubber/polystyrene-vinylpyridine copolymer. The result indicated that the elastomer can be self-repaired at 85 °C for 6 h, and its repair efficiency can reach 83 ± 10%. The well-dispersed O-CNOs reduced the intermolecular chain interaction forces, and the polymer chain mobility was enhanced, thus improving the self-healing ability. Moreover, the functionalized O-CNOs provide more abundant carboxyl groups, which enhanced the internal hydrogen bonding to improve the mechanical properties. The as-prepared polymer also shows good shape memory assisted self-healing performance. This unique molecular network design not only provided a new network construction approach for the development of new rubbers to replace vulcanization but also blazed the way for the manufacturing of advanced elastomers with both excellent mechanical properties and self-healing ability.

## Figures and Tables

**Table 1:** Component polymers ratio table.

Samples	PB-COOH	PS-b-P4VP	Zn(AC)_2_	Zn^2+^:COO^−^:VP	m_O-CNOs_	m_O-CNOs_/m_total_
R1	5 g	0.46 g	0.24 g	1:2:2	-	-
R2	5 g	0.92 g	0.24 g	1:2:4	-	-
R3	5 g	1.84 g	0.24 g	1:2:8	-	-
R2-0.5%	5 g	0.92 g	0.24 g	1:2:4	30.8 mg	0.5%
R2-1%	5 g	0.92 g	0.24 g	1:2:4	61.6 mg	1%
R2-2%	5 g	0.92 g	0.24 g	1:2:4	123.2 mg	2%

**Table 2:** T_g_ value of different samples.

Samples	R1	R2	R3	R2-0.5%	R2-1%	R2-2%
T_g_	87 °C	92 °C	100 °C	85 °C	81 °C	74 °C

## Data Availability

The data presented in this study are available on request from the corresponding author.

## Supplementary Materials

The following supporting information can be downloaded at: https://www.mdpi.com/article/10.3390/polym14224879/s1, Figure S1: Self-healing effect of a rectangular R2-0.5 occurred at room temperature (R.T.); Figure S2: Self-healing effect of a U-shaped R2-0.5 occurred at room temperature (R.T.).
