# Supplementary material for: A Metal Coordination-Based Supramolecular Elastomer with Shape Memory-Assisted Self-Healing Effect
Source: Polymers (Basel). 2022 Nov 12;14(22):4879. doi: 10.3390/polym14224879 (PMC9694331; doi:10.3390/polym14224879)
Supplement: Supplementary file 1 [file polymers-14-04879-s001.zip › polymers-1975986-supplementary.pdf]

Supporting Information

A Metal Coordination-based Supramolecular Elastomer with Shape Memory-assisted Self-healing Effect

Fang Xie[1]†, Zhongxin Ping[2]†, Wanting Xu[1], Fenghua Zhang[2], Yuzhen Dong[1], Lianjie Li[1], Chengsen Zhang[3], and Xiaobo Gong[3]*

[1] School of Materials Science and Engineering, Harbin Institute of Technology at Weihai, Weihai 264209, China
[2] National key laboratory of science and technology on advanced composites in special environments, Harbin Institute of Technology, Harbin 150080, P. R. China
[3] School of Naval Architecture and Ocean Engineering, Harbin Institute of Technology at Weihai, Weihai 264209, China

*Author to whom correspondence should be addressed.
†These authors contributed equally to this work.

Additional Results:

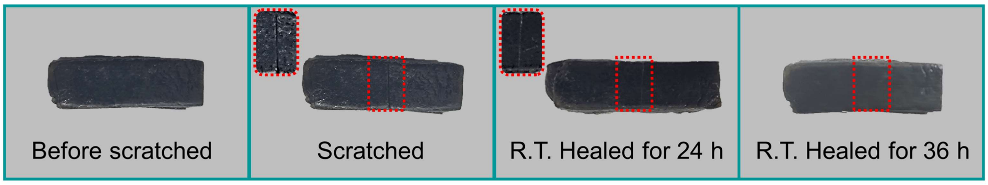

Figure S1. Self-healing effect occurred at room temperature (R.T.).

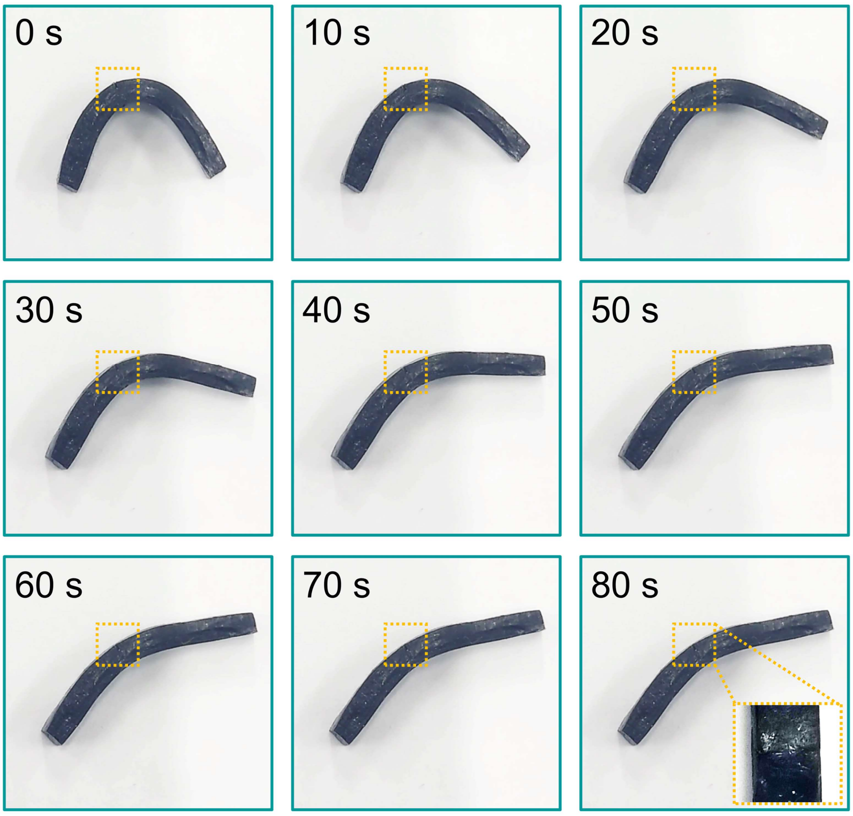

Figure S2. Self-healing effect occurred at room temperature (R.T.).
